# Use of Hospital‐at‐Home Services for Injectable Chemotherapy for Patients With Multiple Myeloma in France in 2019 and 2020: A Real‐World Nationwide Study Based on the French Hospital Discharge Database

**DOI:** 10.1002/jha2.70144

**Published:** 2025-09-11

**Authors:** Laure Vincent, Anne‐Sophie Jannot, Hakima Mechiche, Ulysse Rodts, Gaëlle Désaméricq

**Affiliations:** ^1^ Département d'hématologie clinique CHU de Montpellier Hôpital Saint Eloi Montpellier France; ^2^ Banque Nationale de Données Maladies Rares (BNDMR), AP‐HP Paris France; ^3^ HeKA, INRIA Paris, Inserm, Centre de Recherche des Cordeliers Université Paris Cité Paris France; ^4^ Amgen SAS Courbevoie France; ^5^ KanopyMed Clapiers France

**Keywords:** cancer treatment, chemotherapy at home, hospital‐at‐home, multiple myeloma, outpatient department

## Abstract

**Background:**

Some injectable medicines introduced recently allow patients with multiple myeloma (MM) to receive their chemotherapy at home. This study aimed at describing adult patients with MM receiving injectable chemotherapy via hospital‐at‐home (HAH) services and outpatient hospital units (OHUs) in metropolitan France in 2019 and 2020, analyzing the factors influencing HAH use, and evaluating the geographic variations and the evolution over time of HAH use by these patients during the study period.

**Methods:**

Real‐world data from the French Hospital Discharge Database (PMSI) were analyzed.

**Results:**

In total, 2169/9278 patients (23.4%) received at least one HAH chemotherapy injection. These patients were diagnosed more recently (mean ± standard deviation = 25.1 ± 19.6 vs. 31.6 ± 21.8 months), and lived in larger and wealthier cities (59,000 vs. 41,000 inhabitants; €23,300 ± €5300 vs. €21,700 ± €4100) and closer to their follow‐up hospital (18.7 ± 18.4 vs. 31.3 ± 31.2 km) than patients exclusively treated in OHUs (*p* < 0.001). Receiving bortezomib and carfilzomib, and the first chemotherapy dose in 2020, were the most significant factors associated with HAH use (odds ratio [95% confidence interval]: 6.12 [5.40–6.96], 2.01 [1.69‐2.39], and 1.81 [1.57–2.09], respectively, *p* < 0.001). HAH use increased between 2019 and 2020 (patients, +23%; administrative departments, +25%), likely related to the COVID‐19 pandemic. However, HAH use remained limited overall and exhibited inter‐regional variability. Infection‐related hospitalizations remained stable.

**Conclusions:**

Receiving chemotherapy injections at home is feasible and safe, but further development and equitable access are essential to enhance patients’ quality of life and reduce costs.

## 1 Introduction

Multiple myeloma (MM) is a cancer characterized by the infiltration of bone marrow by clonal plasma cells, leading to anemia, hypercalcemia, renal failure, lytic bone disease, and pathological fractures [1]. With a crude incidence rate of 8.0 per 100,000 persons at risk in France, close to the average of 7.9 per 100,000 in the European Union (EU‐27) [2], MM is the second most common hematological malignancy [3, 4]. In 2018, the median age at diagnosis of MM in France was 70 years for men and 74 years for women [5].

Over the past decade, significant advances in understanding the disease biology, defining the prognosis, and developing novel therapies for both newly diagnosed and relapsed MM have resulted in substantial improvements in overall survival and quality of life (QoL) for patients [1, 6, 7]. Although the ultimate goal of treatment is to provide a cure [8], MM currently progresses towards chronicity, resulting in an increase in the number of patients remaining on treatment for long periods. In 2018, about 17,000 patients with MM received chemotherapy in France (French Hospital Discharge Database [*Programme de Médicalisation des Systèmes d'Information, PMSI*]); effectively managing these patients remains a significant challenge for hospitals.

Some injectable medicines introduced in recent years, however, allow patients with MM to receive their chemotherapy at home. This hospital‐at‐home (HAH) approach has been promoted as an alternative to delivering treatment in outpatient hospital units (OHUs), reducing costs and improving patients’ QoL [9]. However, despite France's reputation as an egalitarian country with a low level of social inequality, geographic disparities in medical care remain significant [10, 11]. The provision of HAH oncological chemotherapy services has been shown to be heterogeneous across the French territory, with four regions (Île‐de‐France, Limousin, Provence‐Alpes‐Côte d'Azur, and Rhône‐Alpes) representing 83% of all HAH activity [12]. To obtain more accurate data concerning MM, we compared the characteristics of patients with MM who had received injectable chemotherapy using a HAH service versus an OHU in France in 2019 and 2020, and analyzed the factors influencing HAH use. Other objectives were to evaluate the geographic variations and the evolution over time of HAH use by these patients during the study period, and to assess the impact of the COVID‐19 pandemic.

## 2 Methods

### 2.1 Study Design and Setting

This observational, retrospective, nationwide study was based on the analysis of real‐world data from the PMSI, which includes anonymous and standardized administrative and medical data for every public and private hospital visit or stay in France.

Patients were included from January 01, 2019 and followed up until December 31, 2020. In addition, some historical data were collected during a baseline period defined as the three years prior to the first dose of chemotherapy received via HAH or in an OHU during the study period.

### 2.2 Ethics

In accordance with French law, the study was registered on the French Health Data Hub (HDH) platform on January 04, 2023. Anonymous data were extracted from the PMSI database according to the MR‐006 reference methodology published by the French data protection agency (*Commission Nationale de l'Informatique et des Libertés, CNIL*).

### 2.3 Participants

Patients with MM as the main diagnosis (i.e., those allocated International Classification of Diseases 10th Revision [ICD‐10] code C90.0 when hospitalized) and who had at least one record of receiving chemotherapy via a HAH service or in an OHU in 2019 or 2020 in a hospital providing HAH services were eligible. A hospital was considered to provide HAH services if at least 10 patients with MM were treated in a HAH setting, dependent on this hospital, during the study period. This threshold was used to limit the risk of re‐identification of patients in apparently anonymous databases, in accordance with the French Directorate for Research, Studies, Evaluation and Statistics (i.e., *Direction de la recherche, des études, de l’évaluation et des statistiques [DREES]*) [13]. Patients aged less than 18 years at the date corresponding to the first HAH‐based or OHU‐based treatment of interest during the study period, those living outside metropolitan France, those who had no possibility of being offered HAH therapy, and patients with any missing data of interest for the study (see “Variables, data sources and procedures” below) or data with extreme values were excluded.

Patients were followed up from the index date—defined as the first discharge date for MM occurring within the inclusion period—to the end of 2020.

### 2.4 Variables, Data Sources, and Procedures

Chemotherapy received in OHUs was identified as an intervention for medical, surgical, and obstetrical diseases (*Médecine, chirurgie et obstétrique, MCO*), coded as 28z07z in the *Groupe homogène de maladies (GHM)*, for which admission and discharge dates were the same. HAH chemotherapy corresponded to interventions with the code 05 as the main mode of care (*Mode de prise en charge principal, MPP*). Hospitalizations for infection were identified as MCO hospitalizations with an ICD‐10 code starting with the letter “B”. Characteristics of the patients and their treatments (age, gender, comorbidities reflected by the Charlson score [14], history of hospitalizations, history of the disease, and history of daratumumab, carfilzomib and bortezomib injections during the baseline period), and data on the hospitals where patients were treated (offer of HAH services reflected by records of HAH chemotherapy for at least 10 patients during the study period, and driving distance between the center of the patient's home postcode area and the hospital) were collected from the PMSI. Data on the locations where patients lived (number of inhabitants and median income of the population in the postcode area) were collected from the French National Institute of Statistical and Economic Studies (*Institut National de la Statistique et des Etudes Economiques [INSEE]*) for the baseline period.

The codes used for programming, including those used for the patient selection and extraction processes, variable definitions, individual analyses, and table construction, were reviewed by the lead statistician and two internal statisticians to limit coding errors. Consistency of the extracted data (e.g., prevalence) was checked against values published in the literature.

### 2.5 Study Size

The number of adult patients with MM as the main diagnosis and at least one chemotherapy injection managed by a hospital providing HAH services in metropolitan France during the study period determined the sample size.

### 2.6 Statistical Methods

Most analyses were descriptive. Qualitative variables were expressed as numbers and percentages, and quantitative continuous variables were expressed as means and standard deviations (SD). Between‐group comparisons were performed using a *t*‐test for continuous variables and the Chi‐square test for binary variables. Moreover, the association of the variables defined above with the use of HAH services for injectable chemotherapy was assessed using logistic regression on a binary classification (at least one HAH dose of injectable chemotherapy vs. no HAH chemotherapy for each patient during the study period) of data from randomly selected patients representing 90% of the study population. Model assumptions were verified using data for the remaining 10% of the study population, and a receiver operating characteristic (ROC) curve was generated using various threshold settings (patients considered as “no HAH” if less than 50%, 20%, or 10% of their chemotherapy injections were received using a HAH service). Analyses were performed using the R software (v3.5) and RStudio [15]. Statistical significance was set at 5%.

## 3 Results

**FIGURE 1:**
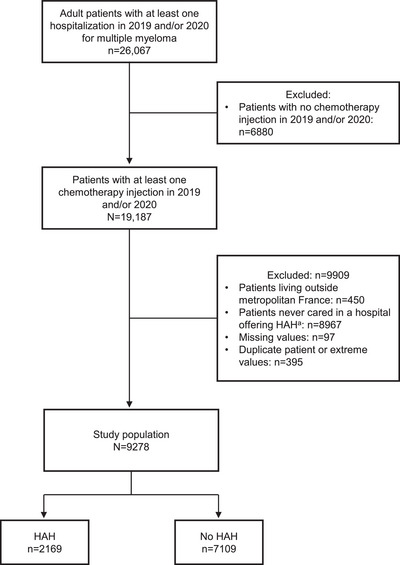
Flow Chart of the Study. Abbreviation: HAH, hospital‐at‐home. ^a^A hospital was considered as providing HAH services if at least 10 patients with MM were treated in a HAH setting dependent on the hospital during the study period.

Among the 26,067 patients hospitalized at least once for MM in 2019 or 2020, 19,187 patients had at least one chemotherapy injection. After applying the exclusion criteria, 9278 patients managed in a hospital with records for at least 10 patients who received HAH chemotherapy injections during the study period were included (Figure 1).

### 3.1 Characteristics of the Study Population

Characteristics of the study population are presented in Table 1. Around one‐quarter of the patients (*n* = 2169/9278; 23.4%) received at least one HAH chemotherapy injection in 2019 or 2020, representing 27,987 HAH interventions. Within the study period, most patients had their first chemotherapy injection in 2019 (*n* = 6196/9278 patients, 66.8%), and bortezomib was the most frequently administered treatment (*n* = 3777/9278 patients, 40.7%). In comparison with patients who did not use a HAH service, those who received at least one HAH chemotherapy injection had a more recent MM diagnosis (mean ± SD = 25.1 ± 19.6 vs. 31.6 ± 21.8 months, *p* < 0.001), lived closer to the hospital where they were followed up (18.7 ± 18.4 vs. 31.3 ± 31.2 km, *p* < 0.001), and resided in geographical areas where the number of inhabitants (59,000 vs. 41,000, *p* < 0.001) and the median income (23,300 ± 5300 vs. 21,700 ± 4100 euros, *p* < 0.001) were higher. There were no significant between‐group differences in age and Charlson score.

**TABLE 1:** Characteristics of the study population.

	Total (*N* = 9278)	HAH (*N* = 1953)^a^	No HAH (*N* = 6399)^a^	*p* value
Age (years), mean ± SD	68.7 ± 11.1	68.6 ± 11.2	68.7 ± 11.0	0.778^b^
Gender, *n* (%)				0.574^b^
Female	4240 (45.7)	959 (44.23)	3281 (46.15)	
Male	5038 (54.3)	1210 (55.7)	3828 (53.8)	
Distance from the hospital (km), mean ± SD	28.5 ± 29.4	18.7 ± 18.4	31.3 ± 31.2	<0.001^b^
Number of inhabitants, mean ± SD	45,725 ± 90,988	5938 ± 13,745	4112 ± 5,803	<0.001^b^
Number of subjects living in a city with >10,000 inhabitants, *n* (%)	8301 (89.5)	1996 (24.0)	6305 (75.9)	<0.001^c^
Median income (per €1000), mean ± SD	22,077 ± 4,436	23,348 ± 5,323	21,728 ± 4,132	<0.001^b^
Duration of the disease (months), mean ± SD	30.1 ± 21.4	25.1 ± 19.6	31.6 ± 21.8	<0.001^b^
Charlson score, mean ± SD	5.7 ± 6.2	5.7 ± 2.7	5.6 ± 2.6	0.688^b^
Carfilzomib, *n* (%)	1356 (14.6)	289 (23.8)	926 (76.2)	0.720^c^
Daratumumab, *n* (%)	2848 (30.7)	544 (21.2)	2016 (78.8)	0.002^c^
Bortezomib, *n* (%)	3777 (40.7)	1365 (39.9)	2053 (60.1)	<0.001^c^
Inclusion year, *n* (%)				0.002^c^
2019	6196 (66.8)	1244 (22.3)	4323 (77.7)	
2020	3082 (33.2)	709 (25.5)	2076 (74.5)	

Abbreviations: HAH, hospital‐at‐home; *n*, number of patients; SD, standard deviation.

^a^ Sub‐sample including randomly selected patients representing 90% of the study population.

^b^ *t*‐test (continuous variables).

^c^ Chi‐square test (binary variables).

### 3.2 Factors Associated With the Use of HAH

The multivariable analyses presented in Table 2 show that treatment with bortezomib and treatment with carfilzomib were the two most important factors associated with the use of HAH chemotherapy (odds ratio [95% confidence interval], OR 95% CI  =  6.12 [5.40–6.96] and 2.01 [1.69–2.39], respectively, *p* < 0.001), followed by receiving the first recorded HAH‐based or OHU‐based chemotherapy dose in 2020 (OR 95% CI = 1.81 [1.57–2.09], *p* < 0.001). Living in areas with a larger number of inhabitants (per 10,000) and higher median income (per 1000 euros) only slightly increased the probability of receiving chemotherapy at home. On the contrary, there was a weak independent association between only receiving chemotherapy injections in a non‐HAH setting and older age, greater distance from the follow‐up hospital, and longer disease duration.

**TABLE 2:** Impact of the study variables on the use of HAH – univariable and multivariable analyses (*n* = 8352)^a^ ^.^

	Odds ratio [95% CI] (univariable)	Odds ratio [95% CI] (multivariable)
Age	1.00 [0.99–1.00]	0.99 [0.98–1.00]
Distance from the hospital (km)	0.98 [0.98–0.99]	0.98 [0.99–1.00]
Number of inhabitants (per 10k)	1.03 [1.03–1.04]	1.01 [1.01–1.02]
City with >10k inhabitants	1.73 [1.42–2.12]	1.13 [0.90–1.42]
Median income in the residence city (per €1000)	1.08 [1.07–1.09]	1.07 [1.06–1.08]
Duration of the disease (months)	0.98 [0.98–0.99]	0.99 [1.00–1.01]
Carfilzomib	1.03 [0.89–1.18]	2.01 [1.69–2.39]
Daratumumab	0.84 [0.75–0.94]	0.98 [0.86–1.12]
Bortezomib	4.91 [4.40–5.49]	6.12 [5.40–6.96]
Inclusion year 2020	1.19 [1.07–1.32]	1.81 [1.57–2.09]

Abbreviations: CI, confidence interval; HAH, hospital‐at‐home.

^a^ Sub‐sample including randomly selected patients representing 90% of the study population.

**FIGURE 2:**
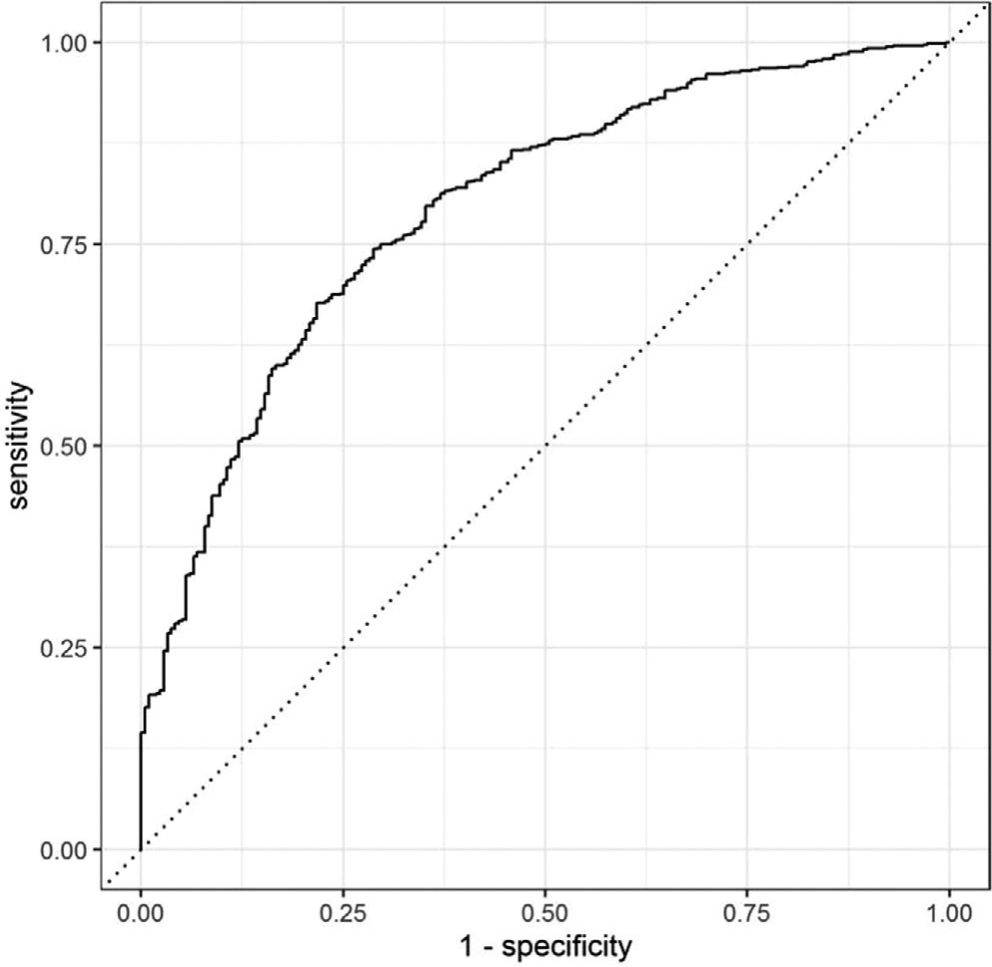
Performance of the multinomial logistic classifier in predicting hospital‐at‐home (HAH) use (*n* = 926 patients). The receiver operating characteristics (ROC) curve was created by plotting sensitivity against 1‐specificity at various threshold settings. These thresholds categorized patients as “no HAH” if less than 50%, 20% or 10% of their chemotherapy injections were delivered by a HAH service. The logistic regression model was developed using data from randomly selected patients representing 90% of the study population (n = 8352 patients) and subsequently applied to the remaining 10% of the study population (*n* = 926 patients) for validation. The resulting ROC curve illustrates the model's discriminatory capacity in identifying patterns of HAH use.

This logistic model was validated on the test dataset composed of data for the remaining 10% of the study population (Table 3 and Figure 2).

**TABLE 3:** Accuracy table for the multinomial logistic classifier (*n* = 926)^a^ **^.^**

	Not HAH, observed	HAH, observed	Percentage of error
**Not HAH, modelled**	**678**	148	17.9%
**HAH, modelled**	32	**68**	32%
**Global percentage of error**			19.4%

Abbreviation: HAH, hospital‐at‐home.

^a^Sub‐sample including randomly selected patients representing 10% of the study population.

### 3.3 Evolution of the Use of HAH From 2019 to 2020

**FIGURE 3:**
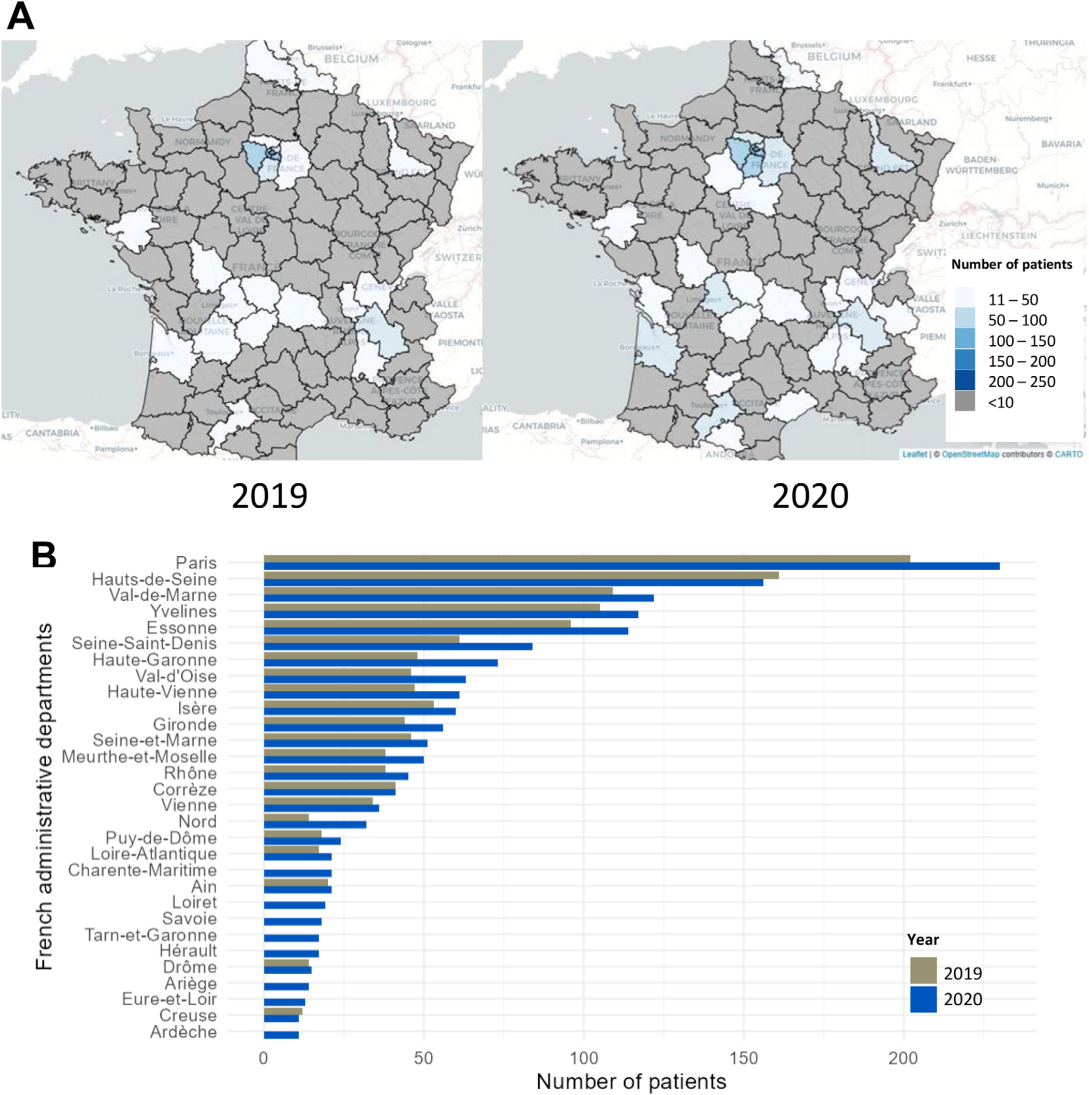
Geographical distribution of patients with multiple myeloma using hospital‐at‐home (HAH) services for injectable chemotherapy in metropolitan France in 2019 and 2020 (*n* = 9278 patients). (A) Choropleth maps illustrate the distribution of patients in French administrative departments, with each color representing a specific value range. (B) Grey bars (2019) and blue bars (2020) depict the number of patients who used HAH services in each French administrative department.

The number of patients with MM treated with injectable chemotherapy in a HAH setting increased by 23% between 2019 and 2020 (*n* = 1411 patients and 13,779 HAH interventions in 2019 vs. *n* = 1735 patients and 14,208 HAH interventions in 2020). Moreover, the number of French administrative departments in which hospitals provided HAH services was higher in 2020 than in 2019 (*n* = 30 vs. 24, Figure 3A,B). Geographical variability was observed, with more than 50 patients with MM using HAH services in only 7 and 12 of the 96 French metropolitan administrative departments in 2019 and 2020, respectively. Most patients who used HAH services (60% in 2019 and 58% in 2020) lived in the Greater Paris area. Overall, the use of HAH services for injectable chemotherapy in MM greatly increased in February 2020, March 2020, and October 2020, i.e., during the first and second waves of the COVID‐19 pandemic in France, whereas the number of OHU visits for chemotherapy injections decreased during these periods (Figure 4A). For the French metropolitan administrative regions, increases in the use of HAH services were observed during the same periods, especially during the first COVID‐19 pandemic wave in Auvergne‐Rhône‐Alpes, Centre‐Val de Loire, Hauts‐de‐France, Île‐de‐France, Nouvelle‐Aquitaine, Occitanie, and Pays de la Loire, and to a lesser extent in Provence‐Alpes‐Côte d'Azur (Figure 4B). Of note, the number of MCO hospitalizations for infections did not increase when the number of patients using HAH services increased (Figure 4A).

FIGURE 4Impact of the COVID‐19 pandemic on the setting used to provide chemotherapy injections to patients with multiple myeloma (MM) in (A) metropolitan France and (B) 12 French administrative regions (patients from Corsica were excluded because of extreme values). The graphs depict the temporal evolution of the monthly number of patients using outpatient hospital unit (OHU, in blue) or hospital‐at‐home (HAH, in red) services for injectable chemotherapy throughout 2019 and 2020. Lockdown periods (from March 17, 2020 to June 1, 2020 and from October 30, 2020 to December 14, 2020) are demarcated by vertical dotted red lines. (A) In addition, the monthly number of patients with multiple myeloma hospitalized for infections in MCO (medical, surgical and obstetrical) during the study period is represented by the black line.

**Figure:**
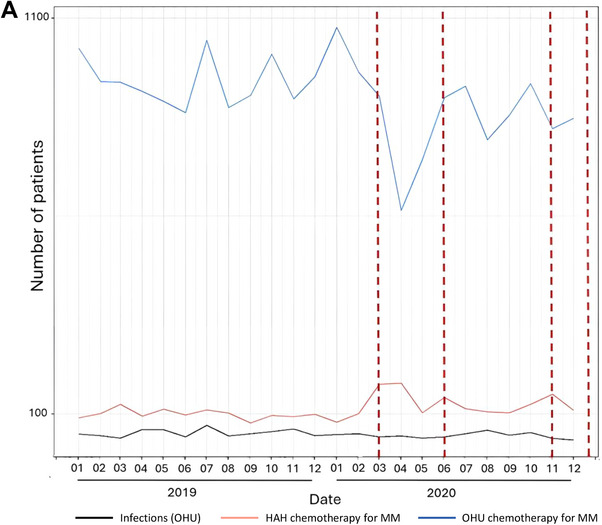

**Figure:**
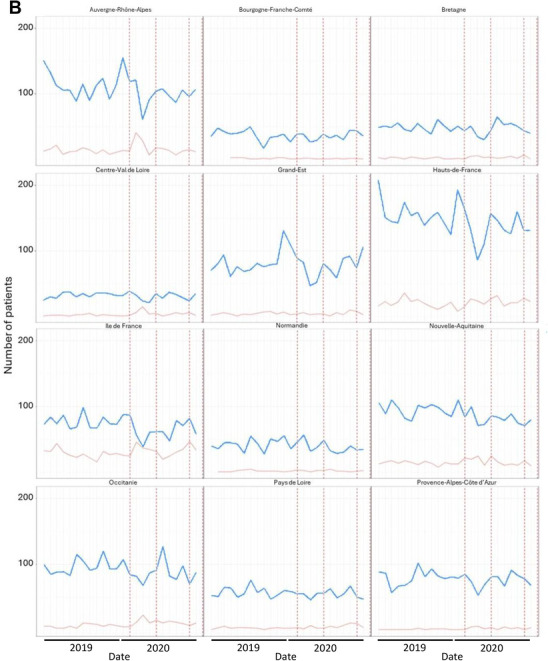

## 4 Discussion

Our study showed that 23.4% (*n* = 2,169/9,278) of patients with MM managed in a hospital with HAH records for at least 10 patients during the study period received at least one HAH chemotherapy injection in 2019 and 2020. Compared with patients exclusively treated in OHUs, patients who received at least one HAH chemotherapy injection were diagnosed more recently, more commonly received bortezomib than other molecules, lived in larger and wealthier cities, and resided closer to the hospital where they were followed up. However, patients’ age was similar in both groups. Treatment with bortezomib and treatment with carfilzomib were the two most important factors associated with the use of HAH for chemotherapy administration, followed by receiving the first recorded dose of chemotherapy in 2020, likely influenced by the COVID‐19 pandemic. The increase in the use of HAH between 2019 and 2020 was evident from both the number of patients receiving chemotherapy at home (+23%) and the number of administrative departments with centres offering HAH treatment (+25%). However, the development of HAH services remained limited both in general and geographically, with inter‐regional variability.

The results of this study are consistent with previous findings [16]. Moreover, as found in a study analyzing anti‐cancer chemotherapy in general [12], most HAH chemotherapy injections for MM in France were performed in the Île‐de‐France administrative region. The variability in French HAH services and in patient selection criteria for administering immune checkpoint inhibitors at home for cancer patients has also previously been highlighted [17, 18]. These findings underscore the need for standardized guidelines and practices for chemotherapy injections at home.

During the COVID‐19 pandemic, OHU chemotherapy was the most frequently used method, even in 2020. The increase in the number of patients with MM using HAH services for injectable chemotherapy in February and March 2020 was smaller than the decrease in the number of patients using OHUs for chemotherapy injections during the same period. This discrepancy may have been due to patient deaths (e.g., COVID‐19‐related deaths), although the number of patients receiving injectable chemotherapy at the hospital after lockdown did not seem to be much lower than before lockdown. In addition, as the existence of HAH services is not among the data available in the PMSI, we considered a minimum number of patients with HAH records (*N* = 10 patients to limit the risk of reidentification) as a surrogate for the availability of HAH services in a given center. Therefore, the number of patients who received chemotherapy at home may have been underestimated, unlike the number of patients managed in OHUs. Treatment interruption may be another hypothesis explaining this discrepancy. Indeed, the duration of MM treatment depends on the patient's age and the therapeutic goal; the treatment could have been interrupted in patients with a well‐controlled disease (elderly or younger patients treated for several years). A few doses of chemotherapy could also have been missed in the early period of the COVID‐19 pandemic, when the uncertainty was maximal and the lockdown very strict, either because of the patient's fear of coming to the hospital or by the physicians' decision to postpone some injections in order to either avoid having the patient come to the hospital or prevent further immunodepression due to the treatment. Furthermore, chemotherapy could have been interrupted due to an ongoing SARS‐CoV‐2 infection, regardless of its severity. For other diseases requiring infusions, studies from Spain and Italy reported therapy interruptions during the COVID‐19 pandemic due to patients’ fear of infection or reorganization of the infusion centers [19, 20]. Despite potential missed doses, our study showed the high level of reactivity of French hospitals and medical centers in implementing HAH services for MM patients receiving injectable chemotherapy during this unprecedented period.

Patients with MM are at high risk of infections due to the disease, treatment, and comorbidities [1]. Our study showed that the number of MCO hospitalizations for infections did not increase with the use of HAH, indicating the safety of performing chemotherapy injections at home. The reduction in visits to the hospital could even have decreased the COVID‐19 transmission risk during the pandemic. Besides, HAH may also reduce costs, as previous studies have shown that home administration of bortezomib is cost‐effective and also preferred by patients compared to hospital administration [21, 22]. The potential cost savings of administering injectable chemotherapy at home could reduce the high economic burden of MM in France [23].

More recent data from the PMSI for all cancers combined show that the number of patients receiving anti‐cancer chemotherapy at home is still growing in France (+10.4% in 2023), although inter‐regional variability remains [24]. The development of HAH services is expected to continue, with projections and perspectives outlined by the *Cercle de Réflexion en Immuno‐Oncologie* [25]. Standardized multidisciplinary recommendations for chemotherapy injections in hematology in HAH settings need to be established, similar to those already available for HAH immunotherapies for solid cancers [26].

Future studies should explore the OHU/HAH ratio per hospital and the percentage of HAH versus OHU interventions for each administrative department to identify areas for improvement and ensure equitable access to HAH services in France.

### 4.1 Strengths and Limitations

Our study population was representative of patients with MM, notably in terms of the mean age at inclusion and the use of bortezomib as the most commonly administered treatment [23, 27, 28]. Combining a proteasome inhibitor and an immunomodulatory drug is currently one of the most effective approaches for newly diagnosed, transplant‐eligible, MM patients [1, 4]. The replacement of bortezomib with carfilzomib in these combinations is currently being evaluated, with promising early results that may lead to an increase in reported carfilzomib use in future analyses. Furthermore, the validity, quality, and reliability of the data from the PMSI have been shown to be high and are becoming more reliable due to their importance for both hospital budgets and their use in research [29]. Since 2005, the systematic recording of home‐based cancer chemotherapy has been mandatory in the PMSI [12]. However, patients’ biological and clinical data, which are factors often determining the indication for home care, are not included in the PMSI. Additional studies, e.g., from local registries, are needed to further describe patients with MM using HAH services to receive their injectable chemotherapy, including specific hematological variables such as myeloma stage, lactate dehydrogenase levels, and cytogenetics, as well as the Eastern Cooperative Oncology Group (ECOG) performance status.

Patients with missing data or data with extreme values (2.6%) were excluded, which minimized the information bias. However, several potential biases remained. First, access to only the postcode instead of the full postal address of the patients may have affected the accuracy of distance calculations from hospitals. Likewise, median incomes for the patients’ home postcode area were used because exact data on patient income were not available. Moreover, as mentioned above, the existence of HAH services is not specified in the PMSI, requiring the use of surrogate data. In addition, data were not weighted by the number of inhabitants, which may have influenced the results. Furthermore, our analysis could not explain why the increase in the number of patients with MM using HAH services for injectable chemotherapy during COVID‐19 waves was smaller than the decrease in the number of patients using OHUs for chemotherapy injections during the same period; hypotheses (treatment interruptions, deaths) have been formulated above. Despite these limitations, the study provided valuable insights into the use of HAH services for patients with MM in France.

## 5 Conclusion

This study is representative of patients with MM who receive care at hospitals that offer HAH services. The increase in HAH use during the COVID‐19 pandemic demonstrates its feasibility and safety as an alternative to traditional hospital‐based care. Our findings also underscore the need for further development of HAH services and for equitable access to these services in France. Future efforts should focus on addressing geographic disparities and enhancing collaboration between healthcare providers to optimize the delivery of HAH services, which may improve patients’ QoL and reduce healthcare costs.

## Author Contributions

LV, ASJ, UR, HM, GD designed the research study/contributed to the design, planning, conduct of the study; UR analysed the data; LV, ASJ, UR, HM, GD interpreted the data; LV, ASJ, UR, HM, GD discussed the results; LV, ASJ, UR, HM, GD critically reviewed the manuscript. All authors reviewed the manuscript and approved the final version.

## Disclosure

In accordance with French law, anonymous data were extracted from the French Hospital Discharge Database [*Programme de Médicalisation des Systèmes d'Information, PMSI*] according to the MR‐006 reference methodology published by the French data protection agency (*Commission Nationale de l'Informatique et des Libertés, CNIL*). The MR‐006 reference methodology does not require individual information to be provided to the patients, nor consent to be obtained. However, a dedicated information related to projects based on PMSI data must be published on the website of the data controller, as well as an information notice detailing clearly to the patients how to exercise their data subject rights for access, rectification, or opposition.

## Ethics Statement

In accordance with French law, the study was registered on the French Health Data Hub (HDH) platform on 04 January 2023. Anonymous data were extracted from the French Hospital Discharge Database [*Programme de Médicalisation des Systèmes d'Information, PMSI*] according to the MR‐006 reference methodology published by the French data protection agency (*Commission Nationale de l'Informatique et des Libertés, CNIL*).

## Consent

The authors have nothing to report.

## Conflicts of Interest

LV: expert boards, bibliographic presentations or writing educational documents for Janssen, BMS, Takeda, Sanofi; funding for conference expenses from Janssen, BMS, Sanofi, Pfizer, Takeda. ASJ: Expert boards for Cemka Eval and Amgen. UR received consulting fees from KanopyMed. HM and GD are employee of Amgen SAS and hold Amgen stocks.

## Data Availability

The patient‐level data used for this study are not publicly available due to privacy restrictions. The datasets generated and/or analysed during the current study are available from the corresponding author on reasonable request. The data that support the findings of this study are available from the study sponsor Amgen upon reasonable request. Qualified researchers may request data from Amgen studies. Complete details are available at http://www.amgen.com/datasharing.
